# Supplementary material for: The Immunomodulatory Effect of IrSPI, a Tick Salivary Gland Serine Protease Inhibitor Involved in Ixodes ricinus Tick Feeding
Source: Vaccines (Basel). 2019 Oct 12;7(4):148. doi: 10.3390/vaccines7040148 (PMC6963187; doi:10.3390/vaccines7040148)
Supplement: Supplementary file 1 [file vaccines-07-00148-s001.pdf]

Supporting Materials :

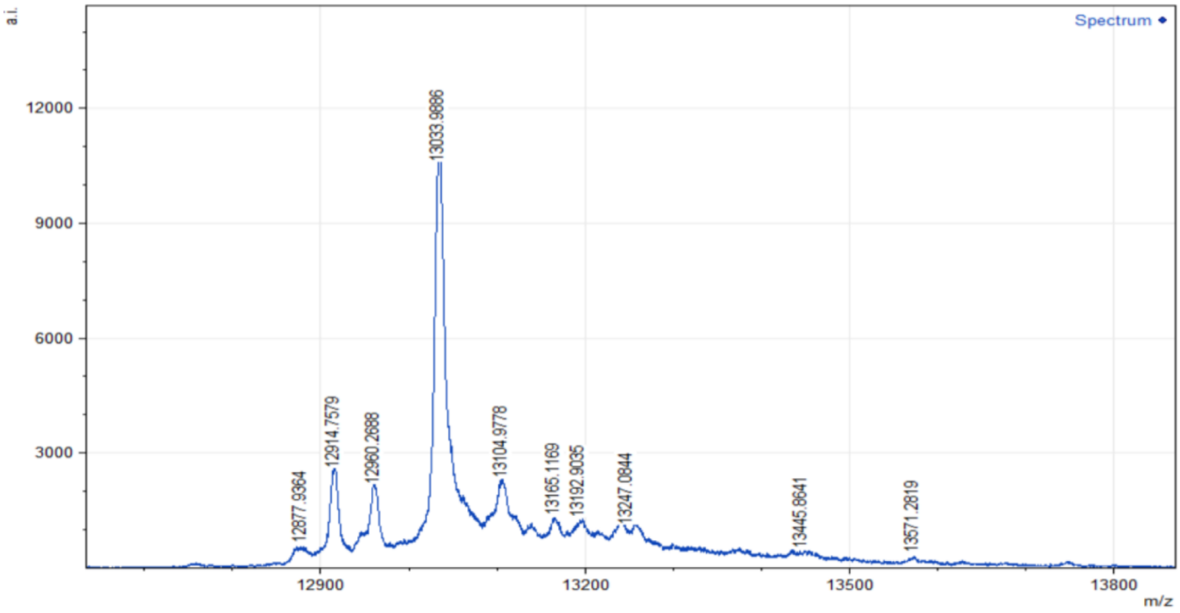

Figure S1. IrSPI MALDI TOF/TOF analysis. A volume of 1 µl at 0.075 mg/mL was deposited on a MTP 384 ground steel target plate with 1 µL2,5-DHB at 25 mg/ml prepared in 50% acetonitrile, 0.1% trifluoroacetic acid in water as a matrix solution. Data were acquired using Flexcontrol software (Bruker-Daltonics, Germany) and shots were recorded in positive ion linear mode.

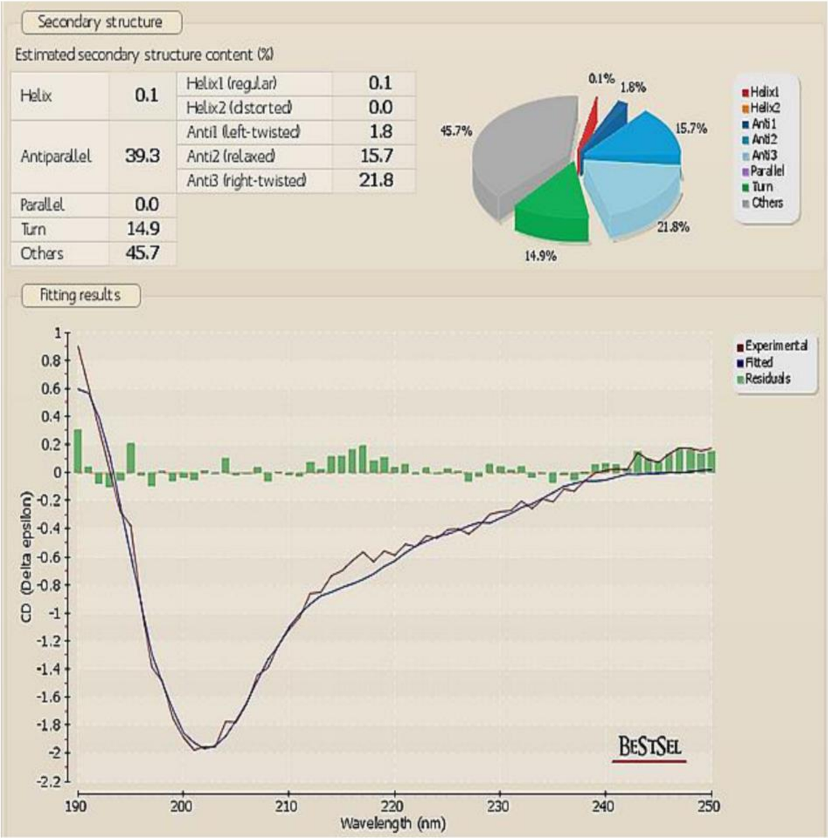

Helix	0.1	Helix1 (regular)	0.1
		Helix2 (distorted)	0.0
Antiparallel	39.3	Anti1 (left-twisted)	1.8
		Anti2 (relaxed)	15.7
		Anti3 (right-twisted)	21.8
Parallel	0.0		
Turn	14.9		
Others	45.7		

Figure S2. Circular dichroism analyses of recombinant IrSPI at 0.075mg/ml. Experiments were performed in the far-UV using an Aviv CD spectrometer model 215 equipped with a water-cooled Peltier unit. Spectra were recorded in a cell width of 1-mm path length in 200µl in far-UV (between 190 to 270 nm) at 25°C. IrSPI and its cognate buffer were successively screened ten times to produce an averaged spectrum, which was corrected using buffer baselines. Data were normalised to residue molar absorption measured in mdeg ($M^{-1}\cdot cm^{-1}$) and expressed as delta epsilon ($\Delta\varepsilon$). BestSel website algorithm treatment was used to predict secondary structures [1].

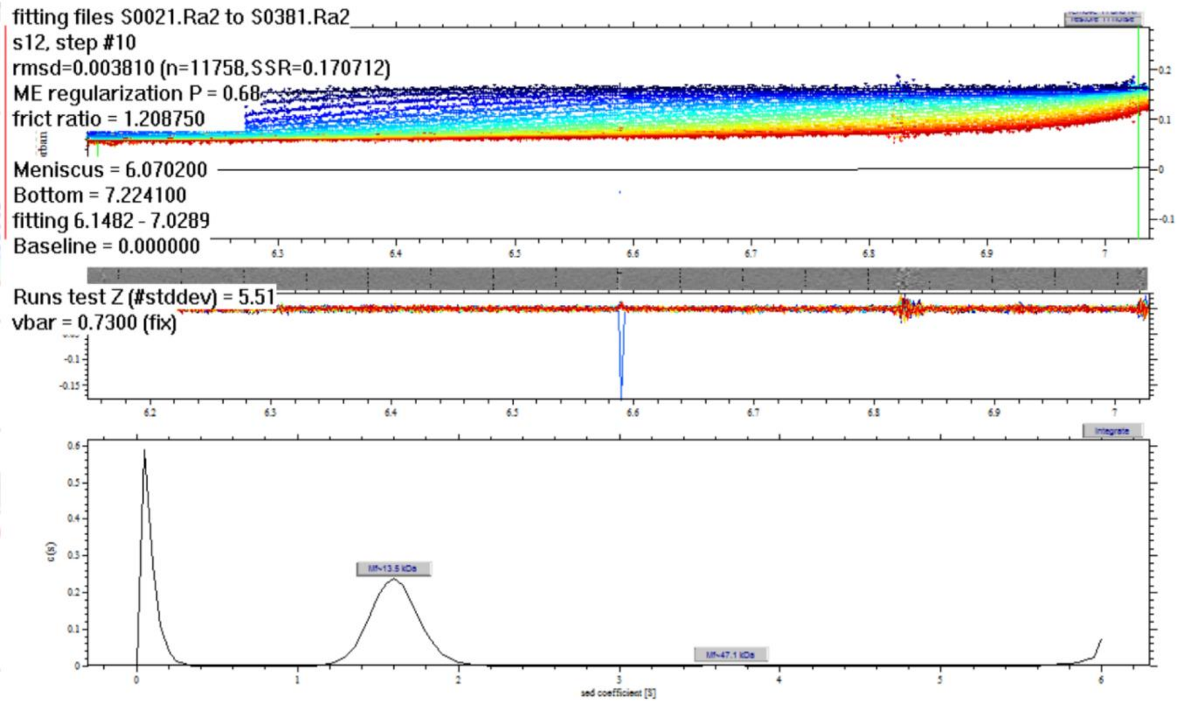

Figure S3. Analytical ultracentrifugation analysis of the recombinant IrSPI at 0.075µg/µl in epoxy double sector cell. The measurement cell was equilibrated for 1.5 h at 20°C in a four-hole AN60-Ti rotor. The sample was spun for 10 h and 400 scans for both Rayleigh interference and absorbance at 280 nm were recorded. Absorbance data were collected at a constant radial step size of 0.001 cm. Partial specific volume of 0.70 and the extinction coefficient ε at 280 nm of 14,350 $L{\cdot}mol^{-1}{\cdot}cm^{-1}$ were theoretically calculated at 20°C from the IrSPI amino acid sequence using Sednterp software (Spin Analytical, Berwick, ME, USA). As a control, the PBS ($-Ca^{2+}$ and $-Mg^{2+}$) density ϱ and viscosity η of 1.0053 $g{\cdot}mL^{-1}$ and 1.018 cP respectively were also determined with Sednterp at 20°C. Data were analysed with Sedfit.

Table S1. Cytokine and chemokine concentrations (pg/ml) in splenocyte supernatants in the presence or absence of IrSPI without ConA stimulation and normalized to standards.

Cytokine/Chemokine	+ IrSPI	- IrSPI
**IFN-γ**	11.2	2.4
**IL-1β**	1.8	2.2
**IL-12p70**	93.2	130.3
**IL-13**	7.7	6.7
**IL-18**	3.6	2.5
**IL-2**	302.7	186.7
**IL-4**	15.3	11.5
**IL-5**	5.9	5.6
**IL-6**	3.9	3.6
**TNF-α**	10.5	10.8
**IL-10**	1.4	0.6
**IL-17A**	0.8	1.2
**IL-22**	0.6	1.0
**IL-23**	0.7	0.7
**IL-27**	0.4	0.0
**IL-9**	4.0	5.4
**GM-CSF**	1.6	0.0
**Eotaxin**	1.8	2.2
**Gro α KC**	15.4	13.1
**IP10**	41.3	27.8*
**MCP1**	44.4	28.8

**MCP3**	22.9	6.3*
**MIP-1α**	1864.4	1642.4
**MIP1-β**	1128.8	834.8*
**MIP2**	51.5	52.6
**RANTES**	293.7	247.9*

* Significant difference as determined using ANOVA (*p*-value < 0.01) after 3 days of exposure to IrSPI. Splenocytes from each of 3 mice were separately evaluated in triplicate for each condition. Results are expressed as the mean for the 3 mice after calculating the mean of triplicate wells for each mouse. Grey lines indicate upregulated cytokines/chemokines in the presence of IrSPI.

Table S2: Cytokine and chemokine concentrations (pg/ml) in splenocyte supernatants in the presence or absence of IrSPI after ConA stimulation and normalized to standards.

Cytokine/Chemokine	- IrSPI	+ IrSPI
**IFN-γ**	7744.2	3849.7*
**IL-1β**	14.3	7.9*
**IL-12p70**	147.2	141.8
**IL-13**	1000.1	248.9*
**IL-18**	157.1	84.8*
**IL-2**	867.3	1293.8*
**IL-4**	719.9	547.2
**IL-5**	48.3	20.1
**IL-6**	134.8	60.9*
**TNF-α**	372.1	200.5*
**IL-10**	24.0	19.7
**IL-17A**	404.4	224.1
**IL-22**	15.4	5.2
**IL-23**	1.3	1.4
**IL-27**	1.4	0.1
**IL-9**	116.7	59.7*
**GM-CSF**	537.2	150.7*
**Eotaxin**	6.8	5.3*
**Gro α KC**	53.3	45.9
**IP10**	1448.3	774.4*
**MCP1**	306.4	276.7
**MCP3**	248.3	202.7
**MIP-1α**	9645.8	8942.3
**MIP1-β**	7711.5	6328.5*
**MIP2**	135.0	109.9
**RANTES**	1633.1	1013.7*

* Significant difference as determined using ANOVA (*p*-value < 0.01) after 3 days of exposure to IrSPI. Splenocytes from each of 3 mice were separately evaluated in triplicate for each condition. Results are expressed as the mean for the 3 mice after calculating the mean of triplicate wells for each mouse. Grey lines indicate upregulated cytokines/chemokines in the presence of IrSPI.

Table S3: Cytokine and chemokine concentrations (pg/ml) in supernatants of activated macrophages in the presence or absence of IrSPI and normalized to standards.

Cytokine/Chemokine	- IrSPI	+ IrSPI
**IFN-γ**	11319.3	10846.1
**IL-1β**	77.7	73.5
**IL-12p70**	261.3	234.1
**IL-13**	164.3	152.3
**IL-18**	360.1	346.9
**IL-2**	38.1	34.8
**IL-4**	22.7	21.2
**IL-5**	58.3	52.8*
**IL-6**	5099.8	4683.6
**TNF-α**	2825.3	2606.5
**IL-10**	1494.8	1440.3
**IL-17A**	7.5	7.5
**IL-22**	6.0	5.6
**IL-23**	5.4	4.9
**IL-27**	203.8	189.3
**IL-9**	34.8	42.9
**GM-CSF**	114.1	109.1
**Eotaxin**	49.7	46.3
**Gro α KC**	4810.4	4302.4
**IP10**	15161.1	14538.4
**MCP1**	16765.5	16234.2
**MCP3**	15040.1	14643.5
**MIP-1α**	5804.3	5707.7
**MIP1-β**	14476.9	14062.4
**MIP2**	23288.1	23188.8
**RANTES**	14591.7	14265.7

* Significant difference as determined using ANOVA (*p*-value < 0.01) after 3 days of exposure to IrSPI. Macrophages from each of 3 mice were separately evaluated in triplicate for each condition. Results are expressed as the mean for the 3 mice after calculating the mean of duplicates wells for each mouse. Grey lines indicate upregulated cytokines/chemokines in the presence of IrSPI.

Reference

1. Micsonai, A. ;Wien, F. ; Bulyaki, E. ; Kun, J. ; Moussong, E. ; Lee, Y. H. ; et al. BeStSel: A web server for accurate protein secondary structure prediction and fold recognition from the circular dichroism spectra. *Nucleic Acids Res.* **2018**, *46*, W315–W322.
